# Research progress on functionalized stem cell therapy strategies in wound healing

**DOI:** 10.3389/fcell.2026.1891271

**Published:** 2026-06-29

**Authors:** Ting Ye, Weihu Zhang, Nanjian Xu, Weihu Ma, Xiaoyin Chai

**Affiliations:** 1 Department of Nursing, Ningbo No. 6 Hospital, Ningbo, Zhejiang, China; 2 Ningbo Clinical Research Center for Orthopedics, Sports Medicine and Rehabilitation, Ningbo, Zhejiang, China; 3 Spine Surgery Center, Ningbo No. 6 Hospital, Ningbo, Zhejiang, China

**Keywords:** functionalization, repair, skin injury, stem cell, therapy, wound healing

## Abstract

The repair of skin injuries, particularly in cases of refractory wounds such as diabetic foot ulcers and severe burns, remains a major clinical challenge. Conventional approaches, including debridement and skin grafting, often fall short of achieving functional regeneration. Although functionalized stem cell therapies have gained interest in recent years, the goal of high-quality functional regeneration has not yet been fully attained. Functionalized stem cells demonstrate unique advantages through multi-target regulation of inflammation, angiogenesis, and tissue remodeling. This review summarizes stem cell sources in skin repair, key functionalization strategies and recent advances in delivery systems that improve stem cell engraftment and repair efficacy. The use of functionalized stem cells represents a promising area of research for skin injury repair. While immune rejection and variable efficacy remain obstacles to clinical translation, multidisciplinary optimization of functionalization strategies may eventually lead to improved management of refractory wounds.

## 1 Introduction

As the largest organ in the human body, the skin is the body’s first line of defense against the external environment, and also undertakes important functions such as sensory interface, immune defense, prevention of fluid loss, and maintenance of homeostasis (Ta and Ea, 2022). After skin injury, normal wound repair usually involves multiple biological processes such as inflammation regulation, cell migration and proliferation, angiogenesis, extracellular matrix deposition and remodeling; once any link is imbalanced, it may lead to delayed healing, persistent chronic inflammation, prolonged infection, or abnormal scar formation (Mamun et al., 2024; Peña and Martin, 2024). Chronic wounds, diabetic foot ulcers, severe burns, and radiation-induced skin damage are the most challenging clinical problems in the field of skin repair. Chronic wounds often remain in a pro-inflammatory state for a long time, accompanied by pathological changes such as biofilm, cell senescence, hypoxia, and impaired tissue regeneration, which seriously hinder normal wound healing (Melnychuk and Fayyazbakhsh, 2026; Shvedova et al., 2026; Vasan et al., 2025; Pearl et al., 2026). Diabetic foot ulcers not only have limited healing rates and high recurrence rates, but are also closely associated with increased risks of infection, osteomyelitis, amputation, and death (Armstrong et al., 2023; Dayya et al., 2022). Burns can cause extensive tissue damage, increased susceptibility to infection, and long-term functional impairment; severely affected patients often face long hospital stays and severe sequelae (Kelly et al., 2022; Alexander et al., 2026). Furthermore, population aging and the rising burden of metabolic diseases are leading to a continuous increase in the incidence of refractory wounds and medical expenditures, placing a heavy burden on patients’ quality of life, family care, and the healthcare system (Mamun et al., 2024; Kim et al., 2021; Marešová et al., 2026). Developing novel treatment strategies that can simultaneously improve inflammatory imbalances, microenvironmental disturbances, and insufficient tissue regeneration is of great significance for skin injury repair.

In recent years, new treatment methods have emerged, such as high-intensity focused ultrasound thermotherapy and natural product therapy. These non-invasive methods have shown excellent results in reducing scarring and promoting burn wound healing (Anastasova et al., 2023; Anastasova et al., 2025). Clinical treatment of skin wounds mainly relies on debridement, anti-infection, decompression, skin grafting, negative pressure therapy, and various dressings. These methods can optimize the local environment and promote wound closure to some extent (Armstrong et al., 2023; Van Rysselberghe et al., 2022; Norman et al., 2022). However, traditional treatments focus primarily on symptom control and wound coverage, with limited intervention in the deeper pathological mechanisms of chronic wounds, such as persistent inflammation, ischemia and hypoxia, cellular dysfunction, and regenerative imbalance (Vasan et al., 2025). For complex wounds, skin grafting and surgery have limitations such as donor site restrictions, high invasiveness, scar contracture, and incomplete functional recovery. Conventional dressings often fail to precisely control the specific pathological processes of chronic wounds (Peña and Martin, 2024; Vasan et al., 2025). Therefore, while traditional strategies can improve some short-term outcomes, they still struggle to achieve high-quality, functional, and low-scarring skin regeneration.

Stem cells self-renew, differentiate into multiple lineages, and secrete cytokines, exosomes, and other bioactive molecules. Via paracrine mechanisms, they regulate inflammation, promote angiogenesis, enhance cell migration/proliferation, and improve the tissue microenvironment (Wang et al., 2025a; Sheykhhasan et al., 2025). Compared to traditional methods that simply cover the wound, stem cell therapy can synergistically intervene in multiple targets of the post-injury pathological process, simultaneously acting on key aspects such as inflammation, fibrosis, hypoxia, and re-epithelialization. Increasing research indicates that stem cell therapy provides new treatment strategies for refractory skin injuries (Alnasser et al., 2025). Building on this, functionalized stem cells can be further enhanced in terms of colonization, survival, and repair efficacy through gene modification, pretreatment, material coupling, or exosome engineering. This article reviews their mechanisms, delivery strategies, and clinical translation progress in skin injury repair.

## 2 Stem cells from different tissue sources and their applications in skin repair

### 2.1 Mesenchymal stem cells

Mesenchymal stem cells (MSCs) are currently the core cell resource in the field of skin injury repair research. MSCs from different sources can effectively accelerate wound healing by promoting cell proliferation and migration, angiogenesis, collagen remodeling, and improving the inflammatory microenvironment (Liu et al., 2022). Adipose-derived MSCs (ADSCs) exhibit significant proliferative, anti-apoptotic, and antioxidant properties in skin repair. Calcium silicate enhances ADSC proliferation, migration, and CXCR4 expression, and improves their reparative efficacy by inhibiting apoptosis-related proteins (Wang et al., 2023). ADSC decellularized extracellular matrix (dECM) patches can significantly promote the proliferation and migration of keratinocytes, endothelial cells, and fibroblasts, and show potential for accelerating wound closure, enhancing angiogenesis, and promoting tissue regeneration in a mouse full-thickness skin defect model (Zhang et al., 2024). Umbilical cord MSCs (UC-MSCs), represented by Wharton’s jelly-derived MSCs (WJMSCs), have advantages such as non-invasive sourcing, low immunogenicity, and high bioactivity. In diabetic wounds, hydrogel-loaded WJMSCs can prolong local cell retention, accelerate the healing process by inducing macrophage polarization towards M2 type, promoting dermal regeneration and type III collagen deposition (Jiao et al., 2022). Pretreatment of UC-MSC supernatant with inflammatory factors can further optimize macrophage biological function and enhance its healing-promoting effect (Liu C. et al., 2022). Bone marrow MSCs (BMSCs) are the best-characterized source, and researchers have extensively described their repair mechanisms. Functionally enhanced BMSCs can accelerate wound healing in mice by upregulating VEGF-A expression and enhancing chemotaxis and paracrine activity (Wang et al., 2024). BMSC exosomes are a promising cell-free therapeutic strategy; for example, exosomes loaded with miR-146a-5p can improve angiogenesis in a high-glucose environment, suggesting broad application prospects in the repair of chronic diabetic wounds (Zhou et al., 2024). ADSC, UC-MSC, and BMSC are the three main cell sources currently used in skin repair research (Shayan et al., 2025; Wang Y. et al., 2025). Although all three have the ability to promote healing, they have different advantages (Mahheidari et al., 2026; Tang A. et al., 2024; Arefnezhad et al., 2024; Li et al., 2024).

### 2.2 Epithelial stem cells

Epidermal stem cells (EpSCs) are a key cell population for maintaining skin homeostasis and regeneration after injury. They are mainly located in specific niches such as the basal layer of the skin and hair follicles. With their excellent self-renewal and differentiation capabilities, they are the core driving force for wound re-epithelialization and skin barrier reconstruction (Hur, 2024). Under physiological conditions, EpSCs are responsible for the continuous renewal of the epidermis. However, when the skin is damaged, progeny cells derived from the interfollicular epidermis (IFE) and different niches in the hair follicles can overcome spatial constraints and be recruited to the wound edge area to jointly initiate the re-epithelialization process and restore skin continuity (Sun et al., 2023). Therefore, the primary value of EpSCs in skin repair lies in their ability to directly provide a cell source for damaged epidermis, accelerating wound closure. However, the therapeutic potential of EpSCs is not limited to cell replacement but also lies in their participation in and improvement of the wound microenvironment. The proliferation, migration, differentiation, and cellular plasticity of EpSCs collectively determine the quality of wound repair; single-cell sequencing and gene knockout technologies are gradually revealing their fine regulatory mechanisms in wound repair (Shi et al., 2022). The biological behavior of EpSCs is affected by factors such as mechanical stress, aging, circadian rhythms, and psychological stress; therefore, clinical applications require simultaneous optimization of the local microenvironment and cell delivery conditions (Tang X. et al., 2024). From a pathway mechanism perspective, multiple signaling pathways are involved in regulating the repair effect of EpSCs: the EGF/EGFR/ERK pathway can upregulate the expression of K19 and Integrin β1, enhance the biological function of EpSCs, and promote re-epithelialization (Li et al., 2021). Fibroblast exosomes can mitigate ROS-induced oxidative damage through the miR-29a-3p/KEAP1/Nrf2 axis, enhance the proliferation, migration, and differentiation capabilities of EpSCs, and upregulate the expression of barrier-related proteins such as Claudin-1 and ZO-1, thereby synergistically improving re-epithelialization, extracellular matrix deposition, and barrier function recovery (Yan et al., 2025). The core application of EpSCs lies in optimizing the quality of re-epithelialization and barrier reconstruction.

### 2.3 Stem cells derived from induced pluripotent stem cells (iPSCs)

**FIGURE 1:**
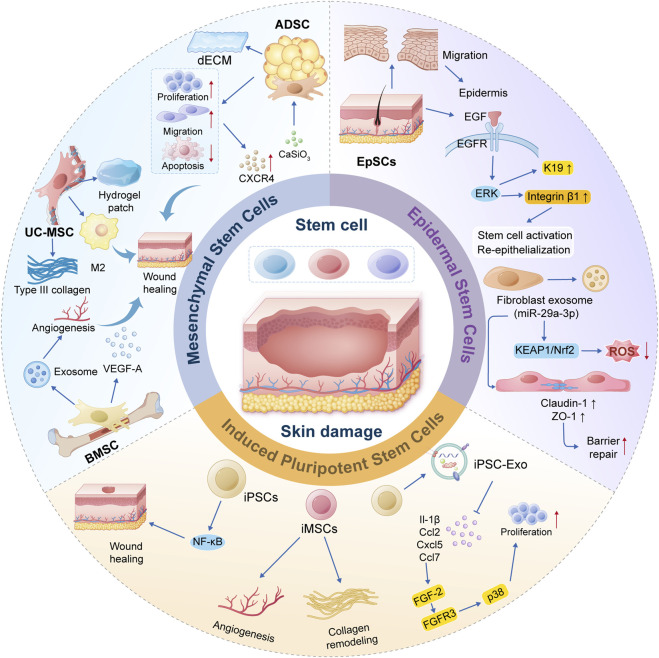
Stem cells from different tissue sources and their applications in skin repair.

Induced pluripotent stem cells (iPSCs) are an important cell source for skin repair and tissue engineering. Their advantages lie in the strong expansion potential and directed differentiation ability of the derived stem cells, while not relying on embryonic origin, thus providing the possibility of avoiding transplant rejection for personalized treatment (Taylor et al., 2011). When keratinocytes induced by iPSCs were transplanted into deep second-degree burn wounds in mice, they significantly reduced the wound area, promoted re-epithelialization, reduced the level of inflammatory factors, and inhibited NF-κB pathway activation, suggesting that iPSC-derived skin lineage cells can directly participate in wound reconstruction (Wu et al., 2023). Similarly, iPSC-derived fibroblasts offer unique advantages for DFU repair. After reprogramming, fibroblasts from both healthy donors and DFU patients show consistent gene expression and function, increased 2D migration, and 3D self-assembled ECM properties that differ markedly from those of primary cells After transplanting this three-dimensional tissue into the wound, the cells can persist in the wound and accelerate the closure of diabetic wounds (Kashpur et al., 2019). HiPSC-induced smooth muscle cells (hiPSC-SMCs), after being delivered via a three-dimensional collagen scaffold, can secrete high concentrations of pro-angiogenic cytokines, increase the number of total macrophages and M2 macrophages in the wound, thereby accelerating wound healing in diabetic nude mice (Gorecka et al., 2020). In summary, iPSCs and their differentiation derivatives have important application prospects in the repair of acute and chronic wounds through multiple mechanisms, including direct participation in epidermal reconstruction, regulation of the immune microenvironment, and promotion of angiogenesis (Ho et al., 2023) (Table 1; Figure 1).

**TABLE 1:** Stem cells from different tissue sources and their application in skin repair.

Types	Characteristics	Mechanisms	References
ADSC	Strong proliferation-promoting, anti-apoptotic, and antioxidant properties; efficacy can be enhanced by calcium silicate	Promotes cell proliferation and migration, angiogenesis, and collagen remodeling Inhibits apoptosis; Calcium silicate treatment upregulates CXCR4 expression	Wang et al. (2023), Zhang et al. (2024), Liu C. et al. (2022)
UC-MSC	Non-invasive sourcing, low immunogenicity, and high bioactivity; hydrogel loading can prolong local retention	Induces macrophage polarization towards M2 type Promotes dermal regeneration and type III collagen deposition; Optimizes macrophage function with inflammation pretreatment supernatant	Jiao et al. (2022), Liu Y. et al. (2022)
BMSC	Most thoroughly researched, with a clearly defined repair mechanism Possesses functional enhancement and numerous exosome strategies	Upregulates VEGF-A expression and enhances chemotaxis and paracrine activity Inhibits TRAF6 and promotes macrophage polarization from M1 to M2 by delivering miR-146a-5p	Wang et al. (2024), Zhou et al. (2024)
EpSCs	Strong self-renewal and differentiation capabilities; a direct driver of skin regeneration; regulated by mechanical stress, aging, and circadian rhythms	Drives re-epithelialization and skin barrier reconstruction; Upregulates K19/Integrin β1 via the EGF/EGFR/ERK pathway; Fibroblast exosomes mitigate oxidative damage through the miR-29a-3p/KEAP1/Nrf2 axis	Hur (2024), Sun et al. (2023), Li et al. (2021), Yan et al. (2025)
iPSC	Somatic cell reprogramming; possesses strong expansion and directed differentiation capabilities; allows for individualized treatment to avoid rejection	Differentiates into keratinocytes, fibroblasts, or smooth muscle cells, directly participating in epidermal reconstruction; Secretes pro-angiogenic factors; Regulates the immune microenvironment	Taylor et al. (2011), Wu et al. (2023), Kashpur et al. (2019), Gorecka et al. (2020)

ADSC, Adipose-derived mesenchymal stem cells; UC-MSC, Umbilical cord mesenchymal stem cells; BMSC, Bone marrow mesenchymal stem cells; EpSCs, Epithelial stem cells; iPSC, Induced pluripotent stem cells.

## 3 Stem cell functionalization strategies

### 3.1 Genetic engineering modification

Genetic engineering aims to overcome functional exhaustion in highly inflammatory pathological microenvironments such as ischemia, high glucose, or severe infection by precisely regulating the gene expression profile of stem cells, which is a core strategy for achieving precise repair of skin injuries. Current research mainly focuses on reshaping the paracrine lineage of stem cells by overexpressing specific transcription factors, signaling molecules, or non-coding RNAs, thereby systematically intervening in the inflammatory, proliferative, and remodeling stages of wound healing.

Enhancing the stress resistance and paracrine activity of stem cells is an important goal of genetic engineering. Using lentiviral vectors to introduce human growth hormone (HGH) into ADSCs, the exosomes secreted by these cells can significantly promote cell cycle progression and accelerate reepithelialization of burn wounds by activating the ERK signaling pathway within fibroblasts (Shao et al., 2025). Correspondingly, knockout of the transcription factor E2F1 (E2F1−/−) reshapes the miRNA profile of ADSC exosomes, significantly activating TGF-β signaling by enriching miR-130b-5p and targeting and inhibiting TGFBR3, thus significantly improving the healing rate of skin defects in mice (Yu H. et al., 2023). Although knockout of E2F1 avoids the introduction of exogenous genes, the loss of transcription factors may lead to uncontrollable changes in the cell cycle regulatory network, thereby affecting the genomic stability of stem cells. In addition, although ADSCs overexpressing hematopoietic prostaglandin D synthase (HPGDS) or IL-10 can improve diabetic wounds by promoting M2 macrophage polarization, whether this long-term anti-inflammatory state will interfere with the body’s normal immune surveillance function still requires further in-depth immunological safety assessment (Ouyang et al., 2022; Zhao H. et al., 2026). In the regeneration of skin appendages, microsphere-based ADSCs carrying HGF and 5α-dihydrotestosterone can activate AKT/ERK signaling to induce sebaceous gland differentiation, achieving functional wound repair (Tao et al., 2022).

In addition, stem cells can be used as bioreactors to load specific miRNAs or circRNAs and deliver them via exosomes. MSC exosomes overexpressing miR-150–5p and miR-93–3p effectively rescued apoptosis by targeting PTEN and APAF1, respectively (Xiu et al., 2022; Shen et al., 2022). However, as natural carriers, exosomes often exhibit low loading efficiency and significant batch-to-batch variability, making it difficult to standardize engineered exosome production like chemical drugs. Regarding angiogenesis, while miR-542–3p and miR-125b-modified stem cell exosomes can promote collagen deposition (Xiong et al., 2023; Guo et al., 2025), over-activated angiogenesis signals pose a risk of inducing abnormal angiogenesis or hemangiomas (Qiu et al., 2024). Furthermore, miR-132 and miR-21-5p-engineered ADSC exosomes achieve a win-win situation of anti-inflammatory and angiogenesis-promoting effects by blocking NF-κB signaling (Ge et al., 2023; Su et al., 2025a), this strong inhibition of key inflammatory pathways may mask potential bacterial infection in wounds, delaying the diagnosis and treatment of infected wounds. In lncRNA research, while H19 and XIST can inhibit pyroptosis and promote fibroblast migration (Yang et al., 2023; Zhu and Quan, 2022), the long chain structure of lncRNAs makes them easily degraded in serum, and their short *in vivo* half-life and low bioavailability have not yet been effectively addressed. Similarly, MALAT1 and SENCR constitute key mechanisms for exosome-mediated angiogenesis by regulating the miR-378a/FGF2 axis and stabilizing the DKC1/VEGF-A axis, but their clinical translation is still limited by the purity bottleneck of large-scale preparation (Pi et al., 2022; Sun et al., 2022).

Researchers are also exploring the immunomodulatory functions of reprogrammed stem cells. While BMSCs overexpressing the chemokine receptor CXCR2 can transdifferentiate into keratinocyte-like cells to accelerate epidermal remodeling (Wang et al., 2025a), the plasticity of stem cells is a double-edged sword. Under specific microenvironments, it remains unclear whether this transdifferentiation carries the risk of reversing into mesenchymal cells or even undergoing epithelial-mesenchymal transition (EMT). In composite scaffold strategies, BMSCs carrying the Jagged1 gene combined with three-dimensional collagen scaffolds have improved cell colonization (Khan et al., 2022), but the interaction between the degradation products of the scaffold material and the genetically modified cells may produce unpredictable immune responses. Furthermore, while IL-1β-prestimulated BMSC exosomes and HMOX1-overexpressing MSC exosomes can promote angiogenesis (Li R. et al., 2025; Cheng et al., 2024), these strategies often rely on complex *in vitro* pretreatment or gene editing procedures, significantly increasing the cost and regulatory complexity of clinical translation. Especially for ADSC exosomes overexpressing HISLA, although they simultaneously correct Th1/Th2 immune imbalance by activating HIF-1α signaling (Zhao W. et al., 2026), the sustained activation of HIF-1α has been shown to be closely related to metabolic reprogramming in various solid tumors, raising concerns about their long-term safety. Finally, while circular RNAs (circRNAs) such as circ-Snhg11 can promote angiogenesis through anti-ferroptosis (Tang T. et al., 2024), their unique structure makes it difficult for current detection methods to completely eliminate immunogenic impurities from their preparation process. Similarly, circ-0001747 and circ-Erbb2ip, by targeting HIF-1α and Nrf1, restored endothelial cell function and reduced ROS levels, respectively, but the long-term stability of their expression and potential off-target effects still require attention (Wang et al., 2025b; Ta et al., 2024).

In addition to traditional viral transduction, emerging non-viral delivery systems are also constantly appearing. For example, lipid nanoparticles (LNPs) co-delivering self-amplifying RNA (saRNA) and E3 mRNA enable long-term stable expression of HGF and CXCL12 in ADSCs, demonstrating superior efficacy compared to traditional transfection in diabetic wounds (Xue et al., 2024). However, the cationic lipid component of LNPs may induce cytotoxicity or excessive inflammatory responses. Furthermore, a surface engineering strategy based on DNA nanofibers significantly enhanced the cells’ ROS clearance capacity and vascular-targeted adhesion by in-situ self-assembling of multivalent fiber structures on the stem cell surface, providing a new approach for physical modification without the risk of gene integration (Wang et al., 2024). However, whether such artificially modified cells will be recognized as foreign bodies and rapidly cleared by the immune system in large animal models remains to be verified. Meanwhile, metabolic click chemistry technology, which couples IL-10 to the surface of MSC-derived nanovesicles, enhances immunomodulatory function but introduces unknown metabolic burdens that may be triggered by exogenous chemical bonds (Ko et al., 2025). Future research should not only focus on validating new targets, but also establish a rigorous safety evaluation system, develop controllable gene switch systems, and formulate quality control standards for engineered exosomes.

### 3.2 Stem cell pretreatment

Stem cell pretreatment strategies induce adaptive changes in stem cells by mimicking *in vivo* stress signals *in vitro*, thereby enhancing their paracrine capacity and tissue repair function. Current research mainly focuses on chemical and drug pretreatment and physical pretreatment.

Regarding cytokine pretreatment, IL-1β prestimulation of BMSC-derived exosomes can regulate the SIRT6/NLRP3 signaling pathway, inhibiting inflammation and promoting angiogenesis (Li R. et al., 2025). Pretreatment of hUC-MSCs with a combination of TNF-α and IL-1β can increase miR-215–5p expression in exosomes, thereby activating the WNK1/p-Smad3/VEGF-A signaling axis and promoting endothelial cell proliferation and angiogenesis (Zhou et al., 2026). Pharmacological pretreatment is also effective. Melatonin pretreatment of BMSCs enhances collagen synthesis, angiogenesis, and antioxidant capacity, while inhibiting inflammation, thereby accelerating tissue regeneration and reducing scar formation (Al-Otaibi et al., 2022). Pretreatment with galangin accelerates diabetic wound healing by reducing neutrophil-associated inflammation, downregulating the IL-17/NF-κB pathway, and promoting angiogenesis and tissue remodeling (Li et al., 2026). Galangin pretreatment of Wharton’s jelly-derived mesenchymal stem cells resulted in exosomes that alleviated oxidative stress-induced fibroblast senescence, reversed SASP, and promoted collagen deposition and angiogenesis (Wen et al., 2026). These different pretreatment strategies all converge on common pathways of anti-inflammation, pro-angiogenesis, and anti-aging. However, most current evidence comes from *in vitro* and animal studies; preclinical data are limited, and long-term safety and optimal delivery protocols have not been determined.

Physical pretreatment strategies do not introduce exogenous genes and have a higher safety profile, playing an important role in stem cell pretreatment as well. Hypoxic pretreatment of BMSC-derived extracellular vesicles promotes cell proliferation and angiogenesis by activating the miR-106b-5p/HIF-1α pathway (Cao et al., 2025). Hypoxic pretreatment of human umbilical cord mesenchymal stem cell-derived extracellular vesicles induces macrophage M2 polarization through the HIF-1α pathway, reducing inflammation and oxidative stress (Su et al., 2025b). Ultrasound stimulation of BMSCs mainly exerts anti-inflammatory, pro-angiogenic, and tissue remodeling effects by enhancing paracrine effects (Tang et al., 2025). Cold atmospheric plasma pretreatment can enhance Fn14 signaling in hair follicle stem cells and activate the Wnt/β-catenin and Sirt1/Nrf2 pathways, thereby promoting the healing of diabetic skin wounds (Zou et al., 2026). Heat shock pretreatment enhances the therapeutic effect of bone marrow mesenchymal stem cells on the healing of diabetic foot ulcers by regulating macrophage polarization, inhibiting inflammatory responses, and promoting fibroblast migration and proliferation (Lin and Lin, 2025). Regardless of cytokines, small molecules, or physical pretreatment, the core logic lies in enhancing the therapeutic efficacy of stem cells or their exosomes by regulating specific signaling axes, and synergistically improving key aspects such as inflammation, oxidative stress, angiogenesis, and cell senescence in the wound microenvironment (Kahrizi et al., 2023).

### 3.3 Exosome/extracellular vesicle engineering

Exosomes, as the core mediator of paracrine function in stem cells, have become an ideal “cell-free therapy” carrier to replace cell transplantation due to their low immunogenicity, lack of tumorigenic risk, and ability to cross biological barriers. However, the therapeutic efficacy of natural exosomes is often limited by the inhibitory effect of the pathological microenvironment and their short half-life. Therefore, exosome engineering aims to reshape their biological characteristics through physical, chemical, or genetic means to achieve precise regulation of skin injury repair.

Altering the culture environment or genetic background of donor cells is the primary strategy to enhance the therapeutic efficacy of exosomes. Preconditioning ADSCs with the flavonoid 3,2′-DHF generates exosomes (Fla-EVs) that activate the MEK/ERK pathway in fibroblasts, and their wound healing efficacy is significantly better than that of the untreated control group (Kim et al., 2023). Similarly, low-intensity ultrasound stimulation, as a non-invasive physical method, can not only significantly increase the exosome production of ADSCs but also enrich them with wound healing-related miRNAs, thereby accelerating healing in diabetic mouse models by promoting re-epithelialization and angiogenesis (Zheng et al., 2023). Regarding drug pretreatment, quercetin and empagliflozin have been shown to enhance the function of umbilical cord MSC exosomes. Exosomes from quercetin-preconditioned cells improve diabetic wound healing by modulating gut microbiota dysbiosis (Wu et al., 2024). In contrast, empagliflozin preconditioning enhances endothelial angiogenesis via the PTEN/AKT/VEGF axis (Wang H. et al., 2025). Studies have confirmed that with age, the proliferative and migration capabilities of apoptotic exosomes (apoEVs) produced by adipose-derived stem cells significantly decline, suggesting that young donors are key to obtaining high-quality exosomes, which also limits the clinical application of autologous aged cell therapy (Yan et al., 2024). Furthermore, excessive physical stimulation may cause cellular stress, which in turn alters the natural composition of exosomes and produces unpredictable side effects.

Directly overexpressing specific genes or loading therapeutic drugs into donor cells is an advanced strategy for endowing exosomes with specific “weapons.” For example, by transfecting antagomiR-15a/16/214 to inhibit the expression of anti-angiogenic miRNAs in ADSCs, the secreted exosomes can more effectively promote angiogenesis and re-epithelialization of diabetic wounds (Ma T. et al., 2023). Regarding drug loading, loading valproic acid (VPA) into umbilical cord MSC exosomes to form the VPA-EXO complex can simultaneously exert the dual effects of inhibiting inflammation and promoting angiogenesis, significantly accelerating wound healing in mice (Mu et al., 2024). In addition, exosomes generated from epidermal organoids derived from iPSCs, due to their rich VEGF and high levels of regulatory miRNAs, have shown great potential in promoting angiogenesis (Kwak et al., 2024). Nonetheless, the safety of gene modification strategies remains a major concern. Lentiviral vector-mediated gene transfection carries the risk of random integration, potentially leading to cell carcinogenesis or genomic instability (Ma T. et al., 2023). Simultaneously, drug loading processes often cause physical damage to the fragile exosome membrane structure, leading to exosome rupture or leakage of contents, thereby reducing their *in vivo* stability and targeting ability (Mu et al., 2024). Notably, some gene modifications may disrupt the natural miRNA balance of exosomes. For example, knocking down lncRNAs GAS5 and MALAT1 in human adipose stem cell exosomes, while not affecting their basic pro-healing function, significantly slows down the healing process, revealing a complex regulatory network within exosomes (Krause-Hauch et al., 2025).

Simply injecting free exosomes often faces the challenges of rapid *in vivo* clearance and uneven distribution. Therefore, developing intelligent delivery systems to maintain high local concentrations and sustained-release effects of exosomes is crucial. The ECM@exo system, which incorporates ADSC exosomes into an acellular matrix hydrogel, gels *in situ* at body temperature, provides sustained exosome release for up to 72 h, and significantly improves wound healing in diabetic rats (Song et al., 2023). Similarly, heat-sensitive porcine acellular dermal matrix hydrogels not only exhibit good biocompatibility but also improve the metabolic microenvironment of diabetic wounds by upregulating the PI3K-Akt pathway (Zhang X. et al., 2026). For special sites such as the cornea, oxidized guar gum self-healing hydrogels, due to their transparency and tissue adhesion, can firmly anchor exosomes to the defect site, promoting corneal epithelial regeneration (Wei R. et al., 2025). 3D bioprinting enables the construction of patient-specific, biomimetic tissue structures that promote wound closure, enhance vascularization, and restore skin integrity in chronic ulcers such as diabetic foot ulcers, pressure ulcers, and venous leg ulcers (Chortova et al., 2026).

Despite the excellent performance of these delivery systems, their limitations cannot be ignored. The degradation rate of hydrogels often fails to perfectly match the tissue regeneration rate; excessively rapid degradation leads to a burst release of exosomes, while excessively slow degradation hinders the ingrowth of new tissue (Song et al., 2023). Furthermore, while decellularized matrices from pigs or bovines can mimic natural ECM, they still pose a risk of triggering xenoimmune responses in the host, especially in applications involving large-area wounds (Zhang X. et al., 2026). Although 3D printing technology enables personalized customization, its high equipment costs and complex fabrication processes limit its widespread adoption in primary healthcare institutions (Ferroni et al., 2023).

**FIGURE 2:**
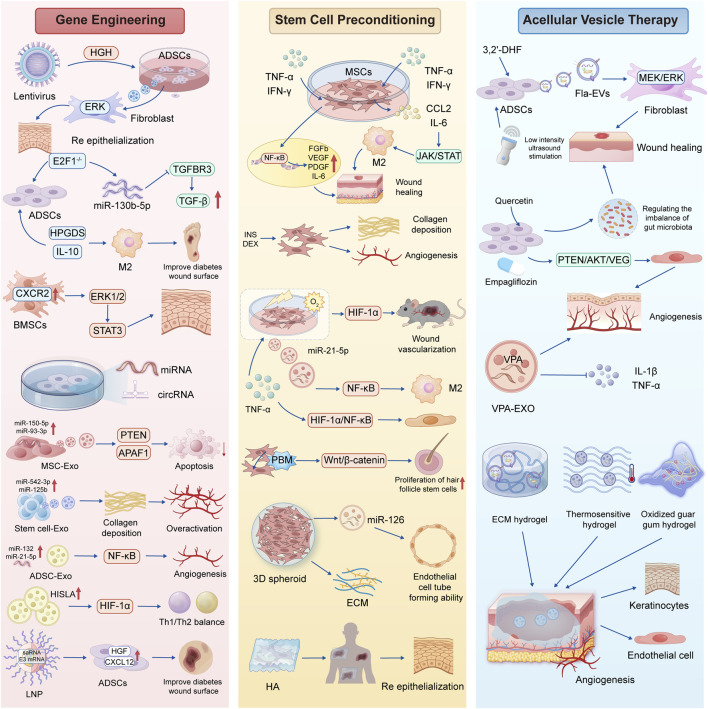
Stem cell functionalization strategies in wound healing.

Promising preclinical results notwithstanding, exosome engineering encounters major obstacles for clinical translation. Firstly, there are issues of heterogeneity and standardization. Exosome characteristics (size, surface markers, cargo) vary significantly with source, passage number, and culture conditions. This variability hinders the establishment of unified quality control standards, such as the MISEV guidelines (Mahheidari et al., 2026). Secondly, long-term safety remains questionable. While exosomes themselves are not tumorigenic, engineered modifications may introduce new risks. For example, although iPSCs produce effective exosomes, undifferentiated iPSCs remaining during preparation may still pose a tumorigenic risk (Kwak et al., 2024). Furthermore, there are bottlenecks in large-scale production. Traditional ultracentrifugation methods result in low yields and are prone to contamination. Although tangential flow filtration (TFF) technology offers a new solution (Chen Y. et al., 2025), maintaining stable cell phenotypes and efficiently collecting exosomes in large-scale bioreactors remains a major challenge for industrial production. Finally, there are species-specific differences in efficacy. Many exosome therapies effective in mouse models show significantly reduced efficacy in large animal models, and single exosome therapies often fall short in the complex microenvironment of chronic human wounds. (Li P. et al., 2025). For instance, a high-glucose environment itself inhibits the uptake of exosomes by keratinocytes and subsequent autophagy activation, thereby weakening the therapeutic effect (Ren et al., 2024) (Figure 2).

## 4 Delivery system and scaffold materials

In therapeutic applications, the clinical translation bottlenecks of direct stem cell transplantation, such as easy loss and low survival rate, make the construction of a delivery system that can simulate the natural extracellular matrix crucial (Sorg et al., 2017). Among various materials, hydrogels have become the core carrier for improving the efficacy of stem cell therapy for skin injuries due to their excellent biocompatibility, highly tunable physicochemical properties, and ability to optimize the wound microenvironment (Keshavarz et al., 2024).

Chitosan, as one of the representative materials of natural polymer hydrogels, has advantages such as biocompatibility, biodegradability, suitable porosity, swelling, and hydrophilicity in chitosan-alginate (CA) composite hydrogels. It can serve as a cell carrier and a platform for delivering bioactive substances. Studies have found that it promotes mesenchymal stem cell-mediated wound healing under hypoxic conditions (Ghahremani-Nasab et al., 2023). In another study, chitosan-polyethylene glycol (PEG) composite hydrogels enriched with MSC exosomes exhibited multiple biological effects *in vitro*, including promoting angiogenesis, regulating inflammation, and enhancing fibroblast migration (Ezati et al., 2025). Polymer hydrogels, with their precisely tunable degradation rates, mechanical strength, and network structures, demonstrate unique advantages in complex wound microenvironments. PLGA-PEG-PLGA (PPP) thermosensitive hydrogels undergo rapid sol-gel transition at approximately 32 °C, exhibiting excellent self-healing properties and biocompatibility after loading with MSC-derived exosomes; in a rat full-thickness skin defect model, they effectively accelerated wound healing by promoting angiogenesis, accelerating myofibroblast differentiation, and reducing inflammation (Wei Z. et al., 2025). In another study, methacrylamide collagen (GelMA) hydrogel was used to load MSC conditioned medium and extracellular vesicles, enabling controlled treatment of acute and chronic wounds by regulating the diffusion and release of EVs, providing a robust delivery solution for maintaining the bioactivity of secretions and ensuring product stability (Doshi et al., 2024). Synthetic polymer hydrogels effectively prolong the retention time and bioavailability of stem cell derivatives in wounds, and are an important carrier strategy for promoting the clinical translation of cell-free therapy. Based on traditional hydrogels, smart responsive hydrogels, by sensing pathological signals such as pH, glucose, reactive oxygen species (ROS), and enzymes in the wound microenvironment, represent a cutting-edge direction for achieving precise and personalized stem cell delivery. For example, a glucose/pH dual-responsive hydrogel designed for the high glucose and acidic microenvironment of diabetic wounds can achieve controlled release of ADSC exosomes through a dynamic triple cross-linking network, promoting M2 macrophage polarization, inhibiting the Notch/NF-κB/NLRP3 pathway, and accelerating angiogenesis and collagen deposition (Zhang et al., 2025). Another study developed a glucose/ROS dual-responsive hydrogel for the intelligent delivery of MSC nanovesicles in diabetic wounds (Du et al., 2024). In burn repair, a dual-layer programmed hydrogel (Dual-Gel) features an inner layer that responds to bacterial hyaluronidase to release photosensitizer-functionalized stem cell nanovesicles for antibacterial purposes, while the outer layer continuously consumes excess ROS to promote tissue regeneration, achieving a triple regulation of antibacterial, antioxidant, and repair-promoting effects (Zhao et al., 2024). Furthermore, an adaptive multifunctional hydrogel can adaptively release MSC-EVs according to the wound condition, preventing rapid clearance and continuously activating repair signaling pathways (Chen X. et al., 2025). Overall, intelligent responsive hydrogels are driving stem cell therapy towards a new stage of proactive sensing and precise regulation.

Researchers have developed various innovative systems for stem cell delivery using 3D bioprinting technology. Gelatin-alginate gradient stiffness 3D-printed scaffolds can simulate the dermal mechanical microenvironment. After loading ADSCs, they significantly promote angiogenesis and wound healing by enhancing their paracrine function (Ma Y. et al., 2023). dECM-GelMA-HAMA composite scaffolds loaded with hADSCs can reduce inflammation, increase percutaneous blood flow, and promote re-epithelialization, orderly collagen deposition, and angiogenesis in rat full-thickness skin defects (Fu et al., 2023). After researchers functionalize hUC-MSC-derived sEVs, the 3D-bioprinted genipin-crosslinked gelatin scaffolds exhibit excellent physicochemical properties, biodegradability, and ECM-mimicking environment, providing a new engineering platform for cell-free therapy (Taghdi et al., 2026). Besides 3D bioscaffolds, natural nanomaterials also play a unique role. β-Chitosan nanofibers (β-ChNF) can self-gel and load ADSCs. The spherical growth of ADSCs secretes more exosomes, and direct application to the wound surface can significantly accelerate epithelialization, granulation tissue formation, and collagen production (Liu Y. et al., 2022). Biological wound dressings prepared from bacterial cellulose membranes (BCMs) combined with BMSCs can promote type I collagen synthesis and angiogenesis by upregulating COL-1 and VEGF-A expression and activating the Notch signaling pathway (Wang et al., 2022). Fiber hydrogel scaffolds loaded with hUC-MSCs can prolong cell survival time at the wound surface, upregulate EGF, TGF-β1, and VEGFA gene expression, and promote re-epithelialization and angiogenesis. The combined therapeutic effect is significantly better than the application of cells or hydrogels alone (Hu et al., 2023). In the field of functionalized composite scaffolds, selenium nanoparticles/chitosan/cellulose nanofiber self-healing hydrogels (SeNPs@CS-CNFs), after loading ADSCs, can scavenge ROS by mimicking GPX activity, promote collagen deposition and angiogenesis in full-thickness defects in diabetic rats, and exert anti-inflammatory effects (Bi et al., 2025). Biosynthetic microsphere technology encapsulates ADSCs in clinical-grade biomaterials, not only protecting cells from damage during cryopreservation, thawing, and injection, but also promoting tissue repair, reducing scar formation, and bringing the type I/III collagen ratio closer to normal skin levels in porcine full-thickness skin defects (Zuo et al., 2023). Scaffold materials, through structural biomimicry, mechanical adaptation, bioactivity modification, and cell protection, are jointly driving stem cell therapy towards higher efficiency and precision. Microneedle patch system, as an emerging transdermal delivery platform, can directly deliver stem cells or their derivatives to the deep layers of the wound by penetrating the stratum corneum, bypassing systemic circulation and minimizing side effects, showing unique advantages in the treatment of diabetic wounds and chronic wounds (Bigham et al., 2025). Hyaluronic acid-based core-shell microneedle patches encapsulate iron-containing MSC-derived artificial nanovesicles (Fe-MSC-NVs) in the core to promote angiogenesis, while simultaneously encapsulating polydopamine nanoparticles (PDA NPs) in the shell to scavenge ROS and inhibit oxidative stress; the two work synergistically to promote M2 macrophage polarization, achieving multiple repair effects of antioxidation, anti-inflammation, and pro-angiogenesis in diabetic wounds (Xu et al., 2025). Another study developed GelMA/PGLADMA core-shell microneedles. The outer shell consists of hydrophilic GelMA loaded with the anti-inflammatory small molecule mangiferin for early and rapid release, while the inner shell consists of hydrophobic PGLADMA loaded with hMSC-derived exosomes for sustained release. Through differentiated degradation kinetics, these microneedles achieve sequential anti-inflammatory and angiogenesis-promoting effects, and significantly reduce scar formation *in vivo* (Lyu et al., 2024).

**FIGURE 3:**
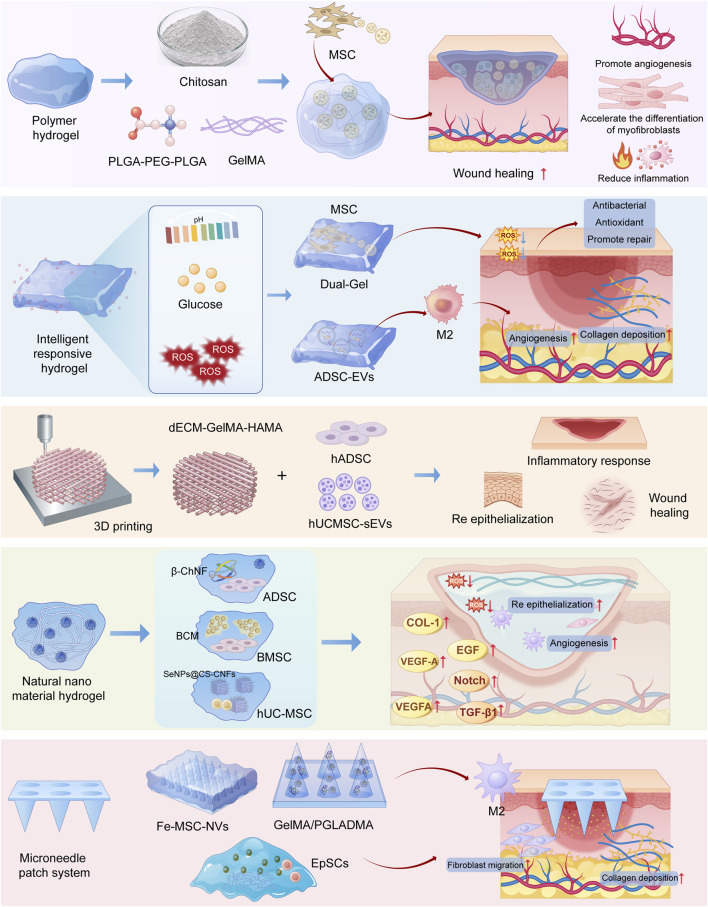
Delivery system and scaffold materials.

In the field of cell patches, bilayer cell patches co-transplant EpSCs with angiogenic ADSCs modified with antagomiR-15a/16/214, significantly promoting the healing of diabetic wounds by enhancing angiogenesis and re-epithelialization (Ma T. et al., 2023). Alginate/ECM/conditioned medium composite patch (AEC patch) can significantly increase secretosome content and prolong release time. *In vitro*, it can effectively stimulate fibroblast migration, proliferation and collagen synthesis. *In vivo*, it can promote cell recruitment, angiogenesis, keratinocyte migration and mature collagen deposition. Over time, it can transform myofibroblast phenotype into fibroblast phenotype, and finally achieve mature angiogenesis (Kwon et al., 2023) (Figure 3).

## 5 Current status of clinical application

At present, most studies on functionalized stem cells for skin injury repair are preclinical, employing strategies such as gene editing, preconditioning, scaffold/hydrogel loading, tissue-engineered skin substitutes, and stem cell-derived exosomes to enhance therapeutic efficacy. Multiple preclinical studies have confirmed their efficacy. Stem cell therapy, represented by MSCs derived from bone marrow, adipose tissue, and umbilical cord, can promote the healing of diabetic foot ulcers (DFU) through multiple pathways, including promoting angiogenesis, regulating immune responses, reducing oxidative damage, and improving matrix reconstruction (Wang B. et al., 2025). Following severe burns, the release of large amounts of pro-inflammatory cytokines such as TNF-α, IL-1β, and IFN-γ into the bloodstream usually triggers systemic inflammatory response syndrome (SIRS), while increased capillary permeability and apoptosis lead to persistent inflammatory stimulation. Pathological dermal and epidermal regeneration disorders delay wound healing, ultimately resulting in hypertrophic scars or even disfigurement, severely impacting patients’ functional recovery and quality of life (Tammam et al., 2023; El-Sayed et al., 2024). In animal experiments, MSCs, with their low immunogenicity and paracrine capabilities, secrete growth factors such as VEGF, HGF, and EGF to promote angiogenesis and collagen remodeling, while simultaneously regulating inflammatory responses (Tong et al., 2016). MiR-153–3p derived from BMSCs-Ex can induce M2 polarization in macrophages, alleviating the inflammatory microenvironment of wounds (Huang et al., 2025). Furthermore, acetoacetic acid pretreatment of ADSCs significantly promotes burn healing by enhancing cell retention and paracrine signaling (Hu et al., 2026), and topological scaffolds can enhance the paracrine function of BMSCs through mechanotransduction and metabolic reprogramming (Zhang Q. et al., 2026). ADSCs loaded with zwitterionic hydrogels can also effectively accelerate healing and reduce scar formation (Yu Q. et al., 2023). Preliminary evidence for clinical translation has emerged, with ABCB5^+^ dermal MSCs as adjuvant therapy for refractory DFU reducing wound area by 59%–67% at week 12, and no treatment-related adverse events (NCT03267784) (Kerstan et al., 2022). Stem cell-derived exosomes have accumulated 12 preclinical studies and 5 clinical trials in this field, suggesting that cell-free functionalization strategies are gradually entering the clinical translation stage and are expected to outperform traditional cell therapies in terms of safety and standardization (Zhang K. et al., 2026).

However, no high-quality clinical studies have yet validated the efficacy of functionalized stem cells in real-world applications. The few existing clinical studies also suffer from problems such as small sample sizes, high heterogeneity, and a lack of standardized protocols. Large-scale randomized controlled trials are crucial for advancing clinical translation (Hu et al., 2018).

## 6 Conclusion

Stem cells from various tissue sources, especially mesenchymal stem cells and their derived exosomes, can regulate inflammation, angiogenesis, and tissue remodeling through paracrine mechanisms. Functionalization strategies such as genetic engineering, pretreatment, and exosome engineering have significantly improved the colonization, survival, and repair efficacy of stem cells in complex wounds. Studies have shown that this strategy, compared to traditional therapies, is more effective in intervening in inflammatory imbalances, microenvironmental disturbances, and insufficient tissue regeneration, precisely regulating the pathological process of wound healing. In particular, the combination of exosome engineering and smart responsive hydrogels provides a safer and more controllable cell-free treatment approach for refractory skin injuries. Preclinical studies have demonstrated its significant efficacy in promoting wound healing, reducing inflammation, and improving tissue regeneration in different injury models. Combining stem cell strategies with physical or phytochemical approaches may further improve outcomes for refractory wounds (Anastasova et al., 2023; Anastasova et al., 2025). Nevertheless, the field remains largely preclinical, and clinical translation confronts multiple challenges.
